# Structure, Regulation, and Significance of Cyanobacterial and Chloroplast Adenosine Triphosphate Synthase in the Adaptability of Oxygenic Photosynthetic Organisms

**DOI:** 10.3390/microorganisms12050940

**Published:** 2024-05-06

**Authors:** Siyan Yi, Xin Guo, Wenjing Lou, Shaoming Mao, Guodong Luan, Xuefeng Lu

**Affiliations:** 1 College of Life Science and Technology, Central South University of Forestry and Technology, Changsha 410004, China; yisy@qibebt.ac.cn; 2 Hunan Provincial Key Laboratory of Forestry Biotechnology, Central South University of Forestry & Technology, Changsha 410004, China; 3 Shandong Provincial Key Laboratory of Synthetic Biology, Qingdao Institute of Bioenergy and Bioprocess Technology, Chinese Academy of Sciences, Qingdao 266101, China; guoxin@qibebt.ac.cn (X.G.); luangd@qibebt.ac.cn (G.L.); lvxf@qibebt.ac.cn (X.L.); 4 College of Live Science, Henan University, Kaifeng 450001, China; 5 Shandong Energy Institute, Qingdao 266101, China; 6 Qingdao New Energy Shandong Laboratory, Qingdao 266101, China

**Keywords:** cyanobacteria, chloroplast, ATP synthase, protein structure, redox ability, enzyme activity, photosynthesis

## Abstract

In cyanobacteria and chloroplasts (in algae and plants), ATP synthase plays a pivotal role as a photosynthetic membrane complex responsible for producing ATP from adenosine diphosphate and inorganic phosphate, utilizing a proton motive force gradient induced by photosynthesis. These two ATP synthases exhibit similarities in gene organization, amino acid sequences of subunits, structure, and functional mechanisms, suggesting that cyanobacterial ATP synthase is probably the evolutionary precursor to chloroplast ATP synthase. In this review, we explore the precise synthesis and assembly of ATP synthase subunits to address the uneven stoichiometry within the complex during transcription, translation, and assembly processes. We also compare the regulatory strategies governing ATP synthase activity to meet varying energy demands in cyanobacteria and chloroplasts amid fluctuating natural environments. Furthermore, we delve into the role of ATP synthase in stress tolerance and photosynthetic carbon fixation efficiency in oxygenic photosynthetic organisms (OPsOs), along with the current researches on modifying ATP synthase to enhance carbon fixation efficiency under stress conditions. This review aims to offer theoretical insights and serve as a reference for understanding the functional mechanisms of ATP synthase, sparking innovative ideas for enhancing photosynthetic carbon fixation efficiency by utilizing ATP synthase as an effective module in OPsOs.

## 1. Introduction

Adenosine triphosphate (ATP) is a primary energy source utilized by cells, and is involved in almost every cellular metabolic process. Additionally, ATP acts as a coenzyme in phosphorylation reactions, and plays crucial roles in RNA, DNA, and protein synthesis [1,2]. ATP synthase, powered by a transmembrane proton motive force (PMF), rotates and catalyzes ATP synthesis using adenosine diphosphate (ADP) and inorganic phosphorus (Pi), serving as the primary pathway for ATP synthesis in cells [3]. ATP synthase can rotate in the opposite direction to degrade ATP, acting as an ATP degradation enzyme [1]. ATP synthase is an ancient, universal, and abundant protein complex. It is well-conserved regarding its functional mechanisms and protein structure [1,4].

Oxygenic photosynthesis converts light energy into nicotinamide adenine dinucleotide phosphate hydrogen (NADPH) and ATP, which drive carbon fixation and serve as the primary energy sources for lifeforms on Earth [3,5]. Enhancing photosynthetic efficiency is a critical focus in modern society for increasing food production, generating clean energy, and obtaining valuable chemicals [6,7,8]. In photosynthetic organisms, ATP synthase is a pivotal component of the photosynthetic system [3]. It considerably affects the light energy utilization efficiency, growth rate, cellular organic accumulation, and stress resistance in plants [9,10,11,12,13], algae [14,15], cyanobacteria [16,17,18,19], and other photosynthetic organisms [20,21]. ATP synthase has been studied for nearly six decades, and has achieved remarkable advancements, particularly with the application of cryo-electron microscopy in structural biology [3,22,23]. These efforts have significantly contributed to our understanding of its structure, functional mechanisms, and its role in regulating cellular energy and metabolic processes. This article comprehensively reviews ATP synthase in cyanobacteria and chloroplasts (in algae and plants), providing an overview of the structure, functional mechanisms, and transcriptional and post-transcriptional regulatory stages that affect cellular ATP synthase activity. Additionally, we have discussed the role of ATP synthase in metabolic regulation and environmental adaptation of oxygenic photosynthetic organisms (OPsOs) and current research on the modification of ATP synthase to enhance photosynthetic carbon fixation efficiency under stress conditions.

## 2. Structure and Functional Mechanism of ATP Synthase in Cyanobacteria and Chloroplast

**Figure 1:**
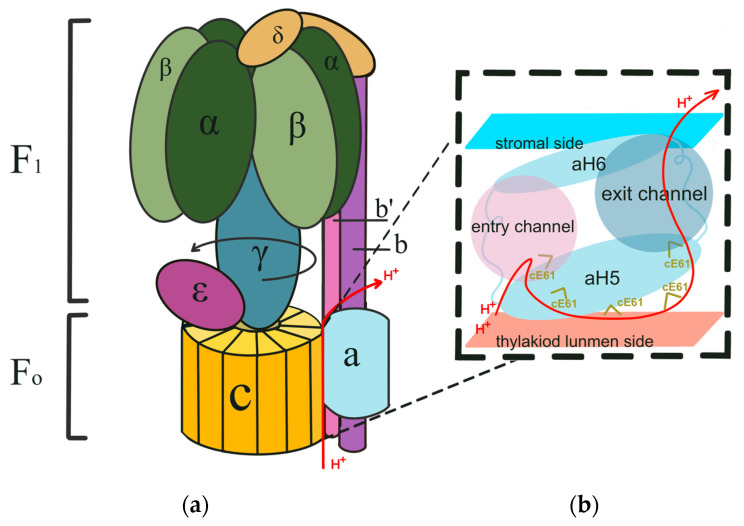
Organization and functional model of ATP synthase in cyanobacteria and chloroplast. (**a**) The model of ATP synthase structure. (**b**) The H^+^ exclusion pathway during ATP synthesis. The rotation direction of the F_1_ complex during ATP synthesis is represented by the black arrow, and the H^+^ exclusion pathway during ATP synthesis is indicated by red arrows. aH5 and aH6, helices 5 and 6 in the a subunit; cE61, the 61st amino acid residue, glutamic acid (E), in the c subunit. The figure is created based on the report by Hahn, A. et al., 2018 [24].

The structure of the chloroplast ATP synthase complex has been reported [24,25], but the complete structure of the cyanobacterial ATP synthase remains unknown [26]. Both cyanobacterial and chloroplast ATP synthase complexes are structurally and compositionally similar to *E. coli* ATP synthase, and are classified as F-type ATP synthases [3,27,28]. They comprise two subcomplexes, F_1_ and F_o_. The hydrophobic part F_o_ is embedded in the membrane, whereas the hydrophilic part F_1_ is exposed on the surface of the thylakoid membrane (Figure 1). The F_o_ complex converts the potential energy of the thylakoid membrane into a rotary force by binding and dissociating with H^+^ ions, acting as the energy-converting motor of ATP synthase. The F_1_ complex utilizes the rotary force generated by F_o_ to catalyze the synthesis of ATP from ADP and Pi, serving as the site where ATP synthase ultimately converts potential energy into biologically usable chemical energy, ATP.

The F_1_ component is composed of five subunits, α, β, γ, δ, and ε; the final structure comprises a total of three α subunits, three β subunits, and one each of the other three subunits, α_3_β_3_γδε (Figure 1a) [3,24], with a molecular weight of approximately 400 kDa. The α and β subunits are arranged in an alternating manner, forming an α_3_β_3_ hexamer structure. The interface between the α and β subunits composes the catalytic site of ATP synthase, with the β subunit contributing to the main portion [3,24]. Depending on the bonding nucleotide, the β subunit can adapt to three different states: βDP (ADP binding), βTP (ATP binding), and βE (substrate-free). The proton gradient drives the rotation of α_3_β_3_, and induces conformational changes in the three β subunits, leading to ATP synthesis from ADP and Pi [29]. The α subunits constitutively bind Mg^2+^ and ATP [24]. In addition to being structural components of ATP synthase, the exact function of α subunits is still unclear. The γ and ε subunits are located at the central axis of ATP synthase [24,27]. Structurally, these two subunits connect the F_o_ and F_1_ complexes. Functionally, they transmit the rotary force generated by the F_o_ complex to the F_1_ complex, thereby driving ATP synthesis. The γ subunit consists of two long α-helices and a disordered region, with the two α-helices forming the central stalk and the disordered region interacting electrostatically with the F_o_-c subunit. The ε subunit is composed of eight reverse parallel β-folds at the N-terminus and an α-helix at the C terminus. The α-helix interacts with the F_o_-c subunit, whereas the β-folds form the stalk and interact with the disordered region of the γ subunit, forming the foundation for the interaction with F_o_-c. The δ subunit acts as a stabilizer and connector between the F_1_ and F_o_ complexes through hydrophobic interactions with the α subunit and the b and b’ subunits of the F_o_ complexes, contributing to the overall structure of ATP synthase [24,25].

The F_o_ complex consists of three subunits, namely, a, b, and c, which form ab_2_c_n_ (Figure 1). In chloroplasts, the c-ring is composed of 14 c subunits [24,25], whereas in cyanobacteria, it consists of 13–15 c subunits [30]. The interaction between the a and c subunits forms the proton channel [24]. This channel is primarily composed of polar and charged amino acids in the a subunit. These polar and charged amino acids render the proton channel hydrophilic, and facilitate the flow of H^+^ through the channel [24]. The 61st amino acid residue in the c subunit is highly conserved with glutamic acid (E) in both cyanobacteria and chloroplasts, which binds to H^+^ on the thylakoid lumen side and deprotonates in the high pH environment outside the thylakoid membrane [3,24,28,30]. This binding and dissociation rotate the c-ring, allowing H^+^ to pass through the proton channel between the a and c subunits and generate torque that rotates the F_1_ subunit, ultimately leading to ATP production. This process converts the potential energy of the thylakoid membrane into chemical energy, specifically ATP, which can be utilized by the cell (Figure 1a,b) [3]. The b and b’ subunits interact with the α, a, and δ subunits, connecting the F_1_ and F_o_ complexes and preventing unproductive rotations of the F_1_ and F_o_ complexes [31,32].

The conservation of ATP synthase subunit sequences varies [33]. The amino acid sequences of the catalytic α and β subunits are conserved across all species. The a and c subunits, which together form the proton pump of ATP synthase, and the γ subunit acting as the energy conveyor for the F_o_ and F_1_ complex, are specifically conserved in cyanobacteria and chloroplasts. The other subunits involved in the connection between F_1_ and F_o_ are not conserved in OPsOs or other organisms. This suggests that chloroplast ATP synthase probably has evolved from cyanobacterial ATP synthase, with the latter being the evolutionary ancestor of chloroplast ATP synthase [28,34,35].

## 3. Biogenesis of ATP Synthase in Cyanobacteria and Chloroplast

### 3.1. Transcription and Translation

**Figure 2:**
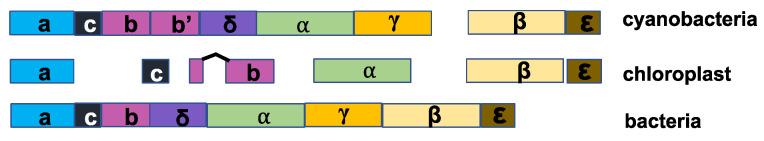
Clustering distribution of ATP synthase-encoding genes in cyanobacteria, chloroplast, and bacteria.

Both cyanobacterial and chloroplast ATP synthases consist of nine types of subunits typically encoded by nine genes [34,35,36]. In most cyanobacteria, these nine genes cluster into two operons, namely γ:α:δ:b:b’:c:a and β:ε [28]. However, in *Synechococcus* 6716, these nine genes are localized in three gene clusters, namely α:δ:b:b’:c:a, β:ε, and γ [33]. In chloroplast, the γ, δ, and b’ subunits of ATP synthase are encoded by the nuclear genome, and the other six subunits encoded by the chloroplast genome are located in two gene clusters, namely a:c:b:α and β:ε, which show a similar distribution as the genes encoding ATP synthase in cyanobacteria (Figure 2) [28]. The genes encoding the nine ATP synthase subunits typically have only one copy in the genome [28,36]. Therefore, the individual subunit synthesis rates must match the assembly of the specific ratio of subunits into the ATP synthase complex. In bacteria, the coding genes are co-transcribed as a single polycistronic mRNA (Figure 2), and the unique stoichiometry of ATP synthase subunits is achieved by regulating the stability and translation rate of this mRNA [37,38,39,40]. The half-lives of mRNA encoding the subunits of ATP synthase vary, with RNase E playing a crucial role in the mRNA inactivation [40,41]. Specifically, *atpB* (subunit a) has the shortest half-life, while the half-lives of *atpE-D* (subunits c, b, δ, α, γ, β) and *atpC* (subunit ε) are 1.2 and 2.5 times longer than that of *atpB*, respectively [41]. The primary and secondary structures of the translation initiation region of the individual subunits significantly impact translational efficiency [37,42,43,44], and the codons and tRNA pools are crucial for the elongation process [38,39]. Similar mechanisms have been observed in chloroplasts. However, there are currently no relevant reports on these regulatory mechanisms in cyanobacteria.

In chloroplasts, multiple protein factors encoded by both nuclear and chloroplast genes play important roles in the splicing, editing, stability, and translation of mRNA encoding ATP synthase subunits. For example, the splicing of *atpF* mRNA involves multiple proteins, such as chloroplast splicing 1 (CRS1), ribonuclease (RNC1), maturase K (MatK), WTF1 (named after “What’s the factor”), Whirly family protein 1 (WHY1), and ATPF editing factor (AEF)1/mitochondrial pentatricopetide repeat 25 (MPR25) [45,46,47,48,49]. Among them, AEF1/MPR25 in *Arabidopsis* is also involved in editing *atpF* mRNA [47]. The biogenesis factor required for ATP synthase (BFA)2 protein binds to the 3′ end of *atpH/F* mRNA, enhancing the stability of *atpH/F* mRNA [50]. Pentatricopeptide repeat-10 (PPR10) binds to the 5′ end of *atpH* mRNA, inhibiting the activity of ribonucleases and acting as a translation initiation activator for *atpH* mRNA [51]. In *Arabidopsis* and maize, (suppressor of variegion) SVR7/ATP4 (named according to The Photosynthetic Mutant Library, essential for the accumulation of chloroplast ATP synthase) and ATP1 participate in the translation of *atpB* and *atpE* mRNA [52,53,54].

Additionally, there can be a reciprocal regulation of transcription and translation among the subunits of chloroplast ATP synthase [55]. In *Chlamydomonas*, the β subunit promotes the translation of the α subunit. In this study, a reporter gene was connected to the *atp-α* promoter region; the results showed that the expression of the reporter gene had no correlation with the protein abundance of the α subunit in the chloroplast, but it exhibited a positive correlation with the content of the β subunit, indicating that the β subunit is a translational activator of the α subunit [55]. The α and β subunits form an αβ dimer, and the “free state” αβ dimer inhibits the translation of *atp-β* mRNA. The γ subunit encoded by the nuclear gene binds to the αβ complex, generating a new protein complex, thereby exerting the inhibitory effect of the αβ dimer on the translation of *atp-β* mRNA. The γ subunit is indispensable for the continuous translation of the β subunit, and it is a translation activation factor for *atp-β* mRNA. It remains unclear if the chloroplast of higher plants applies a similar regulation mechanism as that in *Chlamydomonas*.

### 3.2. Assembling of ATP Synthase in Cyanobacteria and Chloroplast

**Figure 3:**
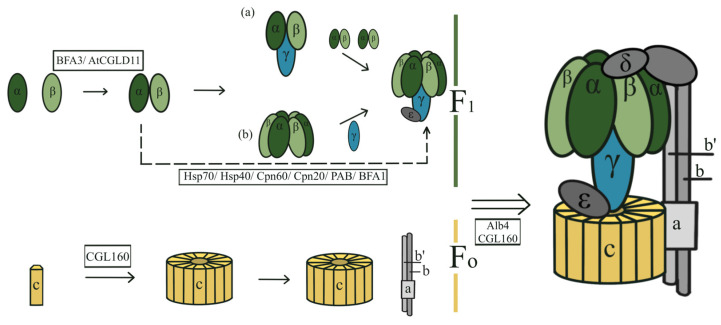
Assembly of chloroplast ATP synthase. BFA: biogenesis factor required for adenosine triphosphate synthase; AtCGLD11: *Arabidopsis thaliana* protein, conserved in the green lineage and diatoms 11; CGL160: conserved only in the green lineage 160; Cpn: chaperonin; Hsp: heat shock protein; PAB: protein in chloroplast ATP synthase biogenesis; and Alb4: Albino 4. Identified ATP synthase intermediates are indicated in colors, and the unidentified fractions are represented in gray. (**a**,**b**), Two hypothesized pathways for the assembly of the F_1_ complex.

Previous studies have shown that the ATP synthase assembly process can be divided into three main stages: (1) assembly of the F_1_ complex, (2) assembly of the F_o_ complex, and (3) linkage of F_1_ and F_o_ to form the complete ATP synthase complex (Figure 3) [56]. In chloroplasts, the initial and crucial step in the assembly of the F_1_ complex is the dimerization of the α and β subunits. There are two hypotheses regarding the subsequent assembly of the chloroplast F_1_ complex: (a) the αβ dimer combines with the γ subunit to form an αβγ trimer, which then interacts with two other αβ dimers to form an α_3_β_3_γ complex [57] and (b) the αβ dimer first interacts with two additional αβ dimers, forming an α_3_β_3_ hexamer, which then binds to the γ subunit, resulting in an α_3_β_3_γ complex [56,58]. However, the intermediate complexes and assembly steps of F_o_ are poorly understood, and further research is needed to explore how the F_1_ and F_o_ complexes are connected and if the δ subunit plays a key role in this process. There are few publications on the assembly of ATP synthases in cyanobacteria.

The assembly of chloroplast ATP synthase involves various accessory proteins that directly affect its proper assembly, abundance, and stability (Figure 3). BFA3/Arabidopsis thaliana protein, conserved in the green lineage and diatoms 11 (AtCGLD11) binds to the β subunit, and plays a vital role in the interaction of α and β subunits, promoting the formation of the αβ dimer. In the BFA3/AtCGLD11-inactive mutant strains, the assembly of the F_1_ complex is inhibited [59,60]. Assembly accessory proteins chaperonin (Cpn)60/Cpn20 play important roles in the proper folding of the γ subunit [61]. Protein in chloroplast ATP synthase biogenesis (PAB) and the γ subunit have a direct protein–protein interaction [62]. In vitro experiments have demonstrated that PAB facilitates the folding of the γ subunit and its binding to the αβ dimer. In the PAB-deficient strain, the chloroplast ATP synthase content decreased by approximately 80%, indicating the significance of PAB in the F_1_ complex assembly. Zhang et al. reported another protein, BFA1, which interacts with both the β and γ subunits and acts as a scaffold to promote the assembly of αβγ trimer [57]. Heat shock protein (Hsp)70 and Hsp40 are also involved in the assembly of the F_1_ complex of chloroplast [61]. The C-terminal domain (CTD) of the conserved only in the green lineage 160 (CGL160) is homologous to that of the ATP synthase protein 1 (Atp1) in prokaryotes (including cyanobacteria) and exhibits partial functional complementarity, playing a crucial role in the assembly of the F_o_-c-ring [11,63]. The N-terminal domain (NTD) binds to the β subunit, promoting the linkage of the F_1_ and F_o_ complexes into an entire ATP synthase complex [11]. The Albino (Alb)4 protein interacts with both the β and b’ subunits, enhancing the stability of the F_1_ complex and participating in the connection of F_1_ and F_o_ complexes [64].

Additionally, the phosphorylation of two tryptophan residues at the NTD of the β subunit is important for the accumulation of chloroplast ATP synthase, and is potentially involved in the assembly of ATP synthase [65]. Besides Atp1, which participates in the assembly of the F_o_-c-ring [11], no accessory ATP synthase assembly proteins have been reported in cyanobacteria, while functional cyanobacterial F_1_ complexes have successfully been expressed in *E. coli* by transforming the F_1_-encoding genes into the bacterium [66,67,68,69,70,71], suggesting that the assembly process of the F_1_ complex in cyanobacteria may be similar to that in *E. coli* and the accessory proteins involved in both systems may possess functional similarities.

Presently, there are few reports on the stability of ATP synthases. In green algae, the chloroplast protease (ClpP) is important in degrading the free subcomplex of ATP synthase [72]; however, the exact degradation pathway is still unclear. In summary, multiple regulatory mechanisms ensure accurate and well-organized transcription, translation, and assembly, leading to the formation of intact and functional chloroplast ATP synthases. However, the corresponding regulatory mechanisms in cyanobacteria remain unknown.

## 4. Regulation Strategy of ATP Synthase Activity in Cyanobacteria and Chloroplast

ATP synthase can degrade ATP when the PMF across the thylakoid membrane falls below a certain threshold to drive the rotation of ATP synthase for ATP synthesis [1,28]. To minimize energy wastage and balance cellular metabolism, the activity of ATP synthase needs to be finely tuned in ever-changing environments. Currently, there are mainly three regulatory strategies for ATP synthase activity in cyanobacteria and chloroplast.

### 4.1. Redox Reaction of the γ Subunit

In photosynthetic ATP synthase, the γ subunit contains a highly conserved insert fragment, approximately 35–40 amino acids [28,73], which is absent in bacteria [28,74], cyanobacteria [26,69] or mitochondria [75]. In chloroplasts, the insert fragment consists of two cysteine (Cys) residues (Cys^199^ and Cys^205^ in spinach chloroplast) with redox properties [28,76]. The redox state of the γ subunit regulates the activity of chloroplast ATP synthase [77]. In the oxidized state, the two Cys residues form a disulfide bond, inhibiting the degradation and synthesis of ATP. After reduction, the Cys residues exist as thiol groups, allowing the restoration of ATP hydrolysis and synthesis. In cyanobacteria, the insert fragment of the γ subunit lacks the central nine amino acid residues, which is unique to chloroplast, and does not contain Cys residues; thus, it does not have redox properties [26,69]. The cyanobacterial ATP synthase activity is independent of its redox state and is, instead, regulated by a small protein known as AtpΘ [16,19,78]. More information about the detailed regulatory mechanism can be found in the subsequent content (in Section 4.3, Cellular ATP Synthase Inhibitor). While the insert fragment plays a crucial role in the ADP-induced inhibition during ATP hydrolysis by cyanobacterial ATP synthase, the underlying mechanism remains unknown [26,66].

The structures of the oxidized and reduced forms of the chloroplast ATP synthase complex deepen our understanding of the redox regulatory mechanisms of ATP synthase activity [24,25]. Compared with the reduced form of ATP synthase, in the oxidized form, the two Cys residues of the γ subunit form a disulfide bond, and the insertion segment forms two hairpins, exhibiting a stable L-shaped distribution [24]. In the reduced form of ATP synthase, the formed disulfide bond breaks, harpin1 disappears, and harpin2 becomes shorter [25]. In both structural forms, harpin2 and the conserved functional domain DELSEED of the β subunit exhibit similar protein–protein interaction [24,25]. Therefore, the stable hairpin structure formed by the insertion segment of the oxidized state γ subunit inhibits the rotation of the F_1_ complex, leading to a decrease in the rotation rate of F_1_ and inhibition of ATP synthase activity. In the reduced state, broken disulfide bonds release more flexibility, and the insertion domain is less resistant to central shaft rotation, resulting in the resumption of ATP synthase activity.

**Figure 4:**
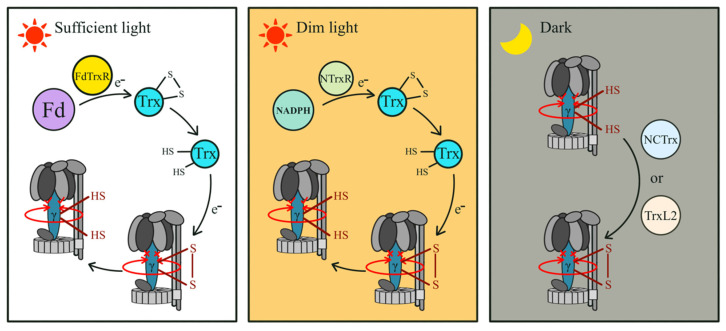
Regulation model of chloroplast ATP synthase activity under different light conditions by controlling the redox state of the γ subunit. Fd, ferredoxin; Trx, thioredoxin; FdTrxR, Fd–Trx reductase; NADPH, nicotinamide adenine dinucleotide phosphate hydrogen; NTrxR, NADPH–Trx reductase; NCTrx, noncanonical Trx; and TrxL2, Trx-like protein 2.

The redox state of the γ subunit is regulated by the thioredoxin (Trx) system (Figure 4). Trx is a thiol/disulfide oxidoreductase, with its active site containing Cys residues, and facilitates the reversible oxidation or reduction of the γ subunit [79,80,81]. Under sufficient light conditions, electrons from photosystem 1 (PS1) are transferred to ferredoxin (Fd), which is reduced. The reduced Fd passes electrons to Trx through Fd–Trx reductase (FdTrxR). Subsequently, the reduced Trx cleaves the disulfide bonds of the γ subunit, activating ATP synthase [79,82,83]. Under dim-light conditions, nicotinamide adenine dinucleotide phosphate hydrogen (NADPH) serves as an electron donor for Trx, mediated by NADPH–Trx reductase (NTrxR) [84]. Under dark conditions, the oxidation of the γ subunit is mediated by two Cys-containing proteins, Trx-like protein 2 (TrxL2) and noncanonical Trx (NCTrx, rich in histidine and Cys) [85]. Additionally, previous studies suggest that the affinity between Trx and the γ subunit has a direct connection with the proton electrochemical potential across the thylakoid membrane (Δ*μ*H^+^). Δ*μ*H^+^ likely induces conformational changes in the γ subunit, increasing the affinity of Trx to the γ subunit and promoting Trx-induced oxidation or reduction of the γ subunit [85,86].

In summary, the state of the γ subunit is closely related to light conditions (Figure 4). Under dark conditions, photosynthesis is inactive, the cellular reductant is low, and the two Cys residues in the γ subunit are oxidized into disulfide bonds by TrxL2 or NCTrx, which blocks the rotation of ATP synthase, inhibiting the reverse rotation and ATP hydrolysis. Under light conditions, photosynthesis is active, cellular reductant is increased, Trx is reduced by NADPH or Fd, and the photosynthetic increase in Δ*μ*H^+^ enhances the affinity of Trx to the γ subunit. Following this, the disulfide bonds in the γ subunit are reduced by Trx as thiol, releasing the blocking effect of the insertion segment, which stimulates the enzymatic activity of ATP synthase for ATP synthesis.

### 4.2. Conformational Change of the ε Subunit

In bacteria, both intracellular ADP/ATP and the PMF across the membrane can induce conformational changes in the ε subunit to regulate the activity of ATP synthase [87]. ADP induces the dissociation of the two α-helices in the CTD of the ε subunit, allowing the extended CTD to bind with the β subunit, thus inhibiting the degradation activity of ATP synthase. The PMF tends to induce the refolding of the extended CTD of the ε subunit, relieving its inhibition on ATP synthase. Some studies suggest that the ε subunit in chloroplasts plays a comparable role in regulating the activity of ATP synthase as that in *E. coli* [88]. However, the structure of the ATP synthase complex of chloroplasts in ε subunit-inhibited state has not been elucidated. Further discussion and studies are needed to illuminate the role and mechanism of the ε subunit in regulating chloroplast ATP synthase.

The ε subunit of cyanobacteria also plays a vital role in regulating the activity of ATP synthase. Unlike in *E. coli*, the CTD of the ε subunit of cyanobacterial ATP synthase alone does not inhibit the degradation activity of ATP synthase [71]. The CTD is not the functional domain of the ε subunit in regulating the activity of cyanobacterial ATP synthase. Instead, the NTD directly participates in the inhibition process. Independent of the CTD, the NTD itself possesses an inhibitory effect on ATP hydrolysis [71]. The crystal structure of the γε complex suggests a tight protein–protein interaction between ε-NTD and the γ subunit [26], which is involved in ADP-induced inhibition during ATP hydrolysis [66], implying that ε-NTD and the γ subunit possibly cooperate to inhibit the degradation activity of cyanobacterial ATP synthase [26,89].

### 4.3. Cellular ATP Synthase Inhibitor

In cells, there are primarily two types of inhibitors for ATP synthase: ADP and protein inhibitors. ADP is a common inhibitor of ATP synthases, and is usually used in structural biology studies on ATP synthases. ADP binds to the catalytic site of the ATP synthase complex and inhibits ATP degradation [90]. There are few reports on the protein inhibitors of ATP synthase in chloroplasts. Here, we focus on the protein inhibitors of cyanobacterial ATP synthase.

In cyanobacteria, photosynthetic and respiratory membranes are integrated, and ATP synthase is involved in both photosynthesis and respiration. During the day, cyanobacteria absorb light and convert it into potential energy via photosynthetic electron transfer, which drives ATP synthase to produce ATP [3,91,92]. At night, cyanobacteria primarily maintain intracellular ATP levels and membrane potential through respiration, and the activity of ATP synthase is considerably lower than that during the day [92,93]. The regulation of cyanobacterial ATP synthase presents unique challenges. Although the activity of ATP synthase in chloroplasts is primarily regulated by the redox state of the γ-subunit to adapt to diurnal changes, the γ-subunit in cyanobacteria lacks redox activity [26,73]. Recent studies have shown that protein inhibitor AtpΘ plays a vital role in regulating ATP synthase activity to adapt to the environmental changes in cyanobacteria [16,19,78]. AtpΘ (approximately 8 kDa) is located in the thylakoid membrane and directly interacts with the ATP synthase complex, inhibiting its degradation activity. The AtpΘ level increases under dark conditions, whereas it is downregulated under light conditions. This regulation pattern is probably related to the transcriptional rate of AtpΘ and the stability of *atpΘ* mRNA. Through changes in AtpΘ levels, the degradation activity of ATP synthase can be controlled to sustain ATP levels in cyanobacteria under dark conditions.

In addition to the abovementioned regulatory mechanisms, chloroplasts also use other mechanisms to regulate ATP synthase activity. For example, under salt stress conditions, ATP synthase forms a dimer via the δ-subunit in spinach chloroplast, maintaining the stability of ATP synthase and the thylakoid membrane [26]. In C_4_ plants, such as maize, there are two forms of α-subunits, namely α and α’, and with an increase in α’ levels under high light conditions, they possibly play a photoprotective role against absorbed excessive light energy [94]. In general, cyanobacteria and chloroplasts employ multiple regulatory systems to adjust the ATP synthase activity and ensure energy and metabolic balance.

## 5. Impact of ATP Synthase Activity on the Energy Metabolism and Environmental Adaptability of Oxygenic Photosynthetic Organisms

### 5.1. Role of ATP Synthase in Regulating the Material and Energy Metabolism of Oxygenic Photosynthetic Organisms

ATP synthase is a crucial photosynthetic membrane complex that significantly affects photosynthesis, carbon fixation, and energy metabolism in photosynthetic organisms [10,95]. Photosynthesis is the process of converting light energy into chemical energy, including reducing power and high-energy phosphate bonds [5]. During the light reaction, the photosystem absorbs light energy, triggering the release of electrons and resulting in photoelectric conversion. Subsequently, through photosynthetic electron transfer, a proton gradient is established across the thylakoid membrane, and nicotinamide adenine dinucleotide phosphate (NADP^+^) accepts electrons to generate NADPH, the main reducing agent in photosynthetic organisms. The proton gradient across the thylakoid membrane drives ATP synthase to pump protons from the thylakoid lumen into the stroma to generate ATP, converting potential energy into biologically usable energy (in the form of ATP) [5,96]. The absorption of every eight photons by photosystem leads to 12 protons accumulated in the thylakoid lumen and four electrons released from H_2_O in PS2 [97]. Depending on the number of c subunit copies in the c ring—13–15 in cyanobacteria and 14 in chloroplast [30]—approximately, 4.33–5.00 protons are needed to synthesize one ATP [98]. Theoretically, this process generates 2.40–2.77 ATP with two NADPH. The low radio of ATP/NADPH is compensated by cyclic electron flow around PS1 to support the downstream carbon reduction [99,100]. The relatively large number of c ring copies in the c ring results in low energy efficiency of ATP synthase and photosynthesis, causing an imbalance of ATP/NADPH level in metabolism [12]. This could represent an evolutionary trade-off between photosynthetic efficiency and adaptability of OPsOs.

Thus, ATP synthase activity has a significant effect on the pH of the thylakoid lumen, redox state of PS1, photosynthetic electron transfer chain, ATP content, and carbon fixation efficiency [10,95]. For example, in the *Arabidopsis* mutant *cfd*, where the γ subunit is consistently in an oxidized state, the activity of ATP synthase decreases, resulting in reduced photosynthetic rate, ATP content, and growth rate [101]. In the *Arabidopsis* chloroplast mutant *atpg*, ATP synthase content decreases significantly, and the mutant exhibits symptoms of chlorosis and is unable to grow photoautotrophically [102]. When the activity of chloroplast ATP synthase decreases, the ΔpH across the thylakoid lumen increases, non-photochemical quenching is enhanced, and photosynthetic linear electron transfer rate, intracellular ATP and NADPH content, and carbon fixation efficiency significantly decrease [9,95]. Conversely, when the ATP synthase activity increases, these physiological parameters undergo opposite changes. For example, in rice, the overexpression of the δ subunit results in a notable increase in the cellular ATP content and activity of chloroplast ATP synthase, and the cyclic electron transfer rate decreases, whereas the linear electron transfer rate and photosynthetic carbon fixation efficiency dramatically increase under high light and high carbon dioxide conditions [103].

Additionally, studies have suggested that ATP synthase is directly involved in gene transcription and expression, and regulates metabolic networks in chloroplasts and cyanobacteria. The α and γ subunits of chloroplast ATP synthase, respectively, participate in the editing of wheat and *Arabidopsis* chloroplast RNA [104,105]. Cyanobacterial ATP synthase interacts with valine-transfer RNA synthetase to affect gene transcription and translation [106]. Overall, ATP synthase directly and indirectly regulates the material and energy metabolism of OPsOs.

### 5.2. Importance of ATP Synthase for Environmental Adaptability of Oxygenic Photosynthetic Organisms

Chloroplast and cyanobacterial ATP synthases have a significant effect on the tolerance to both biotic and abiotic stresses in photosynthetic organisms. For example, in *Synechococcus* 7942, the mutation of F_1_-α-C252Y increases the intracellular activity of ATP synthase, photosynthetic rate, and biomass and significantly enhances the tolerance of the mutant to high temperature and high light stress [17,18]. In plants, the α and β subunits of chloroplast ATP synthase are closely associated with the resistance against biotic and abiotic stresses. The overexpression of the α subunit of chloroplast ATP synthase enhances the resistance of tomatoes to low temperature, high salt, and fungal infections [13]. The overexpression of the β subunit in tomato plants considerably improves light energy utilization, reactive oxygen species scavenging ability, transpiration in the cold, and the tolerance of the mutant to low temperatures [107]. A single nucleotide mutation in the β subunit of chloroplast ATP synthase enhances the tolerance of cucumber to low-temperature stress [108]. These findings suggest that increased ATP synthase activity is a key benefit for OPsOs that survive under stressful conditions. However, in higher plants, decreased ATP synthase activity can be beneficial regarding stress tolerance by triggering photoprotection to modulate the photosystem in response to environmental conditions. For example, under prolonged drought stress conditions, the chloroplast ATP synthase content in watermelon plants significantly decreases, leading to a significant reduction in thylakoid membrane conductance [109]. This phenomenon is considered important for plants to survive long-term drought stress by tuning their photosynthetic system. In *Arabidopsis*, by reducing the presence of accessory proteins necessary for the assembly, the resulting decline in chloroplast ATP synthase activity assists in preserving the stability of the photosynthetic system when exposed to high-temperature conditions [110]. Moreover, the size of the c-ring is closely related to the tolerance of OPsOs to environmental stress [12]. Further investigation is required to explore the precise regulatory mechanism of ATP synthase in stress tolerance and its interplay with the metabolic networks of photosynthetic organisms.

## 6. Conclusions and Perspectives

ATP synthase is one of the main photosynthetic membrane complexes in OPsOs, and its activity is closely related to photosynthetic light energy utilization, biomass accumulation, and the stress resistance ability of photosynthetic organisms. ATP synthase is composed of multiple subunits, and its transcription, translation, assembly, stability, activity, regulation, and interactions with other cellular components are complex. Hence, gaining insights into the regulatory network of ATP synthases in cyanobacteria and chloroplasts, along with their responses to environmental stress conditions, is important for improving photosynthetic carbon fixation efficiency. As previously discussed in Section 5, optimizing the protein amount, activity, and structure of ATP synthase can greatly enhance the photosynthetic carbon fixation efficiency and stress tolerance of OPsOs, thereby exerting a substantial impact on photosynthetic output. This subject is also comprehensively addressed from a different perspective in the recently published paper by Rühle, T. et al. [111].

Modulating ATP synthase to enhance OPsO productivity shows promise, but is still in the early stages. Therefore, it is critical to have deep understanding of its structure, assembly and regulation mechanism for its extensive application in optimizing photosynthetic output. This paper provides an extensive review of the characterization of cyanobacterial and chloroplast ATP synthases over the past decades, covering aspects such as transcription, assembly, structure, and regulation of ATP synthase activity. It also discusses their impact on energy-material metabolism and stress tolerance in OPsOs. However, there are still some crucial issues need to be addressed: (1) the entire structure of the cyanobacterial ATP synthase and its conformational changes in different activity states; (2) the specific and complete transcription, translation regulation mechanism, and assembly process of ATP synthase in OPsOs; (3) the activity regulation mechanisms of ATP synthase under stress conditions; and (4) the functional mechanism of ATP synthase on the stress tolerance of OPsOs, if this effect is directly connected with the material–energy metabolism. The answers to these questions may help us better understand the functional mechanism of ATP synthase in photosynthetic organisms and use ATP synthase as an effective regulatory module to improve the photosynthetic carbon fixation efficiency of photosynthetic organisms, thereby achieving higher economic benefits and generating more clean and usable energy. This, in turn, would benefit the livelihoods of people.

## Data Availability

No new data was created.
